# Síndrome Cardiorrenal Tipo 1 em Região de Baixo Desenvolvimento: Comparação entre os Critérios AKIN e KDIGO, Necessidade de Diálise e Mortalidade

**DOI:** 10.36660/abc.20200097

**Published:** 2021-08-09

**Authors:** Ginivaldo Victor Ribeiro do Nascimento, Heitor Carlos Domingues de Brito, Carlos Eduardo Batista de Lima

**Affiliations:** 1 Universidade Estadual do Piauí TeresinaPI Brasil Universidade Estadual do Piauí, Teresina, PI - Brasil; 2 Faculdade Integral Diferencial Curso de Medicina TeresinaPI Brasil Faculdade Integral Diferencial Curso de Medicina, Teresina, PI - Brasil; 3 Universidade Federal do Piauí TeresinaPI Brasil Universidade Federal do Piauí, Centro de Ciências da Saúde, Teresina, PI - Brasil; 4 Centro de Pesquisa CARDIOLIMA TeresinaPI Brasil Centro de Pesquisa CARDIOLIMA, Teresina, PI - Brasil

**Keywords:** Síndrome Cardiorrenal/complicações, Insuficiência Renal Crônica, Lesão Renal Aguda/normas, Nefropatias/normas

## Abstract

**Fundamento:**

A síndrome cardiorrenal tipo 1 associa-se a maior mortalidade em pacientes com insuficiência cardíaca (IC). No entanto, há escassez de publicações comparando critérios diagnósticos de lesão renal aguda (LRA).

**Objetivos:**

Analisar o perfil clinicofuncional de pacientes com IC e fatores associados a ocorrência de lesão renal aguda (LRA).

**Métodos:**

Estudo de coorte retrospectivo, em hospital terciário de região com baixo desenvolvimento econômico que incluiu pacientes com IC descompensada ou infarto agudo do miocárdio (IAM) recente, sendo avaliadas características clínicas, laboratoriais e ecocardiográficas comparativamente em pacientes com e sem LRA classificada pelos critérios *Acute Kidney Network* (AKIN) e *Kidney Disease: Improving Global Outcomes* (KDIGO). Nível de significância estatística com valor de p < 0,05.

**Resultados:**

Entre 81 pacientes, 61,73% evoluíram com LRA. A média de creatinina foi 1,79±1,0 mg/dL e de ureia 81,5±46,0 mg/dL, sendo maior no grupo com LRA (p < 0,05). Não foi evidenciada relação entre alterações cardíacas e redução da função renal. A doença renal crônica se associou a maior ocorrência de LRA (38% x 3,23% sem LRA, p = 0,001). Não houve diferença do KDIGO com relação ao critério AKIN. Os pacientes que desenvolveram LRA apresentaram maior mortalidade (32% x 9,8% no grupo sem LRA, p = 0,04, com *odds ratio* (OR) de 8,187 e intervalo de confiança 1,402-17,190, p = 0,020).

**Conclusão:**

Nessa casuística de pacientes com IC, a ocorrência de LRA foi elevada e foi fator de risco independente de mortalidade. As alterações cardíacas não se associaram à ocorrência de LRA, e os critérios diagnósticos KDIGO e AKIN apresentaram performance similar.

## Introdução

A síndrome cardiorrenal (SCR) abrange uma diversidade de condições agudas ou crônicas, em que a falência primária de um órgão, que pode ser o coração ou os rins, implica na deterioração secundária do outro.^1^^,^^2^ Aproximadamente um terço dos pacientes com descompensação de insuficiência cardíaca (IC) também podem apresentar um comprometimento agudo da função renal, sendo esta caracterizada a SCR tipo 1.^3^

A presença de disfunção ventricular pode acarretar diversos efeitos negativos sobre a função renal. Ao mesmo tempo, a insuficiência renal pode comprometer significativamente a função cardíaca. Os efeitos diretos e indiretos de cada órgão disfuncional podem iniciar e perpetuar mutuamente uma combinação de desordens.^2^

Nas últimas décadas, o termo lesão renal aguda (LRA) foi revisado, enfatizando-se um processo fisiopatológico progressivo que pode ser perceptível diante de pequenas variações de marcadores de lesão renal, com destaque para a creatinina. Com esse propósito, destacam-se dois critérios diagnósticos: *Acute Kidney Network* (AKIN) e *Kidney Disease: Improving Global Outcomes* (KDIGO).^1^^,^^4^ Este último, que emergiu com a intenção de harmonizar definições e critérios previamente estabelecidos, tem sido largamente utilizado na investigação de IRA e nos cenários de SCR, distinguindo-se também da terminologia usada: piora da função renal.^1^^,^^4^^,^^5^ Na literatura, são poucos os trabalhos nos quais foram avaliados estes critérios de LRA na SCR, sobretudo em países emergentes e em áreas com baixo índice de desenvolvimento econômico.^6^^,^^7^

O objetivo do estudo foi avaliar a incidência de SCR à luz da comparação dos critérios AKIN e KDIGO, bem como avaliar os fatores de risco associados ao desenvolvimento dessa condição patológica e à necessidade de diálise, bem como a mortalidade em pacientes com IC descompensada.

## Metodologia

Realizou-se um estudo observacional de coorte retrospectivo em hospital terciário, referência para atendimento de urgência e emergência, o Hospital de Urgências de Teresina (HUT), tendo como critério de inclusão todos os pacientes admitidos com diagnóstico clínico de IC descompensada em pacientes com cardiopatia prévia, ou devido infarto agudo do miocárdio (IAM) recente e que foram submetidos à realização de exame ecocardiográfico com evidência de fração de ejeção inferior a 55 por cento. Foram excluídos os pacientes com idade inferior a 18 anos, transplantados, que realizavam terapia dialítica crônica e aqueles que não tiveram, no mínimo, duas dosagens de creatinina durante o período de internação.

Esta pesquisa foi revisada e aprovada conforme as normas éticas do Comitê de Ética local, sendo aprovado sob o CAAE n^2^ 54207914.5.0000.5211. Os dados foram coletados através da revisão dos prontuários médicos e dos registros do serviço de ecocardiografia de pacientes internados no período de 1 de janeiro a 31 de dezembro de 2014, cujas variáveis estudadas incluíram: data e diagnóstico de admissão do paciente, idade, gênero, comorbidades (hipertensão arterial sistêmica (HAS), diabetes mel (DM), cardiopatia dilatada, doença cerebrovascular, doença renal crônica (DRC), e hepatopatia, além de características laboratoriais (creatinina, ureia, potássio, bicarbonato, pH), bem como dados evolutivos de melhora clínica com alta hospitalar e óbito.

A definição da LRA foi baseada nas variações de creatinina de acordo com dois diferentes critérios. A classificação proposta pela AKIN apresenta como critérios o aumento dos valores da creatinina em um intervalo de 48 horas e ainda leva em conta a necessidade de terapia de substituição renal. A alteração primária da creatinina sérica maior que 0,3 mg/dL ou seu aumento entre 1,5 a 2 vezes com relação à primeira medida são classificados como lesão em estágio 1. O aumento na faixa de 2 a 3 vezes com relação à primeira dosagem é classificado como estágio 2. Os pacientes que apresentam aumento maior ou igual a três vezes com relação à creatinina inicial, dosagem maior ou igual a 4 mg/dL com aumento abrupto superior a 0,5 mg/dL, ou em início de terapia de substituição renal são classificados como estágio 3.^8^^,^^9^

Segundo a classificação pelo KDIGO, o estágio 1 requer alteração no valor de creatinina maior ou igual a 1,5 do valor basal em um período de sete dias ou de 0,3 mg/dL dentro de um período de 48 horas. O aumento igual ou superior a duas vezes do valor basal é classificado como estágio 2. O estágio 3 corresponde ao triplo de elevação de creatinina com relação ao valor basal, ao valor maior ou igual a 4 mg/dL, ou em início de terapia renal substitutiva.^4^

### Análise estatística

As variáveis contínuas foram expressas em média e desvio-padrão, de acordo com a normalidade da distribuição obtida por meio do teste de Kolmogorov-Smirnov e comparadas pelo teste *t* de Student para amostras independentes (não pareado). As variáveis categóricas foram expressas em proporções e comparadas pelo teste Qui-quadrado de Pearson. Para a análise multivariada, utilizou-se a Regressão de Logística Múltipla (RLM) com razão de chance ajustada. O critério para inclusão de variáveis no modelo logístico foi a associação ao nível de 20% (p < 0,200) na análise bivariada. O critério de significância ou permanência das variáveis no modelo, por sua vez, foi a associação em nível de 5% (p < 0,05). O modelo final de RLM foi ajustado pelo método Enter. O teste de bondade de ajuste (teste de Hosmer e Lemeshow), necessário para a RLM, mostrou que o modelo final é adequado para explicação da variável resposta. A multicolinearidade das variáveis explicativas foi verificada pelo teste VIF (Variance Inflation Factor), adotando-se como ponto de corte para o diagnóstico de multicolinearidade um VIF acima de quatro. Contudo, o teste não detectou multicolinearidade entre as variáveis estudadas. Adotou-se o nível de significância de 5% para todos os testes estatísticos. Os dados foram analisados por meio dos *softwares* R-Project, versão 3.0.2 e Statistical Package for the Social Science (SPSS), versão 20.0.

## Resultados

Nesse estudo, foram incluídos 81 pacientes admitidos com diagnóstico de IC descompensada e/ou infarto agudo do miocárdio (IAM) recente. A caracterização clinicodemográfica da população estudada está demonstrada na Tabela 1. A média de idade dos pacientes foi de 67,02 ± 14,97 anos, 43 deles do sexo masculino (53,09%). O diagnóstico de IC descompensada foi mais frequente em pacientes com cardiopatia prévia e HAS foi a comorbidade mais prevalente.

**Tabela 1:** Caracterização geral de pacientes com insuficiência cardíaca (isquêmica ou não isquêmica) avaliados quanto ao desenvolvimento de lesão renal aguda (N = 81).

Variável	N
**Idade (anos)**	67,02 ± 14,97
Média ± DP	
**Gênero (%)**	43 (53,09)
Masculino	
**Diagnóstico (%)**		
IAM recente	16 (19,75)
IC com cardiopatia prévia	62 (76,55)
IC prévia e IAM recente	3 (3,7)
Outro diagnóstico*	8 (9,88)
**Comorbidades (%)**		
HAS	48 (59,26)
DM	26 (32,1)
DRC	20 (24,69)
Cerebrovascular	7 (8,64)
Hepatopatia	6 (7,41)
Outro	24 (29,63)

IAM: infarto agudo do miocárdio; IC: insuficiência cardíaca; HAS: hipertensão arterial sistêmica; DM: diabetes melito; DRC: doença renal crônica; DP: desvio-padrão.

* Pacientes com IC descompensada e FEVE < 55% sem cardiopatia prévia ou IAM recente, além de arritmias, edema pulmonar hipertensivo e infecção.

A população do estudo foi dividida em dois grupos, formados pelos que evoluíram com LRA (50 pacientes) e sem LRA (31 pacientes). As características clínicas, laboratoriais e ecocardiográficas, bem como os seus desfechos clínicos, são mostrados na Tabela 2.

**Tabela 2:** Caracterização das variáveis clínicas, laboratoriais, ecocardiográficas e desfechos clínicos dos pacientes com LRA e sem LRA de pacientes internados com IC ou IAM (N = 81)

Variáveis	Sem LRA (n = 31)	Com LRA (n = 50)	OR (IC 95%)	p – valor
**Características clínicas**				
Idade (± DP)	64,03 ± 16,08	68,88 ± 14,08	1,02 (0,99-1,05)	0,172

Sexo masculino – n (%)	20 (64,52)	23 (46)	2,13 (0,85-5,37)	0,104	

HAS – n (%)	15 (48,39)	33 (66)	0,48 (0,20-1,20)	0,116
DM – n (%)	8 (25,81)	18 (36)	0,62 (0,23-1,66)	0,339
DRC – n (%)	1 (3,23)	19 (38)	18,4 (2,31-146,10)	**0,001**
Doença cerebrovascular - n (%)	1 (3,23)	6 (12)	4,09 (0,47-35,73)	0,337
Hepatopatia – n (%)	3 (9,68)	3 (6)	0,60 (0,11-3,16)	0,858
Outro – n (%)	7 (22,58)	17 (34)	–	–

**Características laboratoriais (± DP)**				
Ureia (mg/dL)	46,65 ± 25,66	81,52 ± 46,04	1,03 (1,01-1,05)	**0,001**
Creatinina (mg/dL)	1,17 ± 0,76	1,79 ± 0,97	1,02 (1,01-1,04)	**0,002**
Potássio (mEq/L)	4,11 ± 0,75	4,4 ± 1,02	2,76 (1,29-5,91)	0,155
Bicarbonato	24,12 ± 6,11	22,01 ± 4,71	0,92 (0,79-1,07)	0,355
pH	7,417 ± 0,05	7,374 ± 0,1	0,01 (0,01-74,88)	0,069

**Características ecocardiográficas**				
IAM recente – n (%)	8 (25,8)	8 (16)	0,61 (0,22-1,73)	0,507
IC com cardiopatia prévia – n (%)	22 (70,97)	40 (80)	1,83 (0,61-5,51)	0,429
IC prévia e IAM recente – n (%)	1 (3,23)	2 (4)	-	0,999

Fração de ejeção: % (± DP)	35,86 ± 10,79	36,09 ± 10,79	1,01 (0,97-1,05)	0,598

Diâmetro de AE: mm (± DP)	39,51 ± 7,45	39,51 ± 7,45	1,02 (0,96 - 1,08)	0,624

**Espessura miocárdica – n (%)**				
Aumentado	16 (51,61)	21 (42)	0,77 (0,31-1,90)	0,538
Normal	15 (48,39)	29 (58)	Referência	

**Disfunção sistólica do VE– n (%)**				
Leve	1 (3,23)	2 (4)	Referência	0,999
Moderada/grave	30 (96,77)	48 (96)	1,25 (0,18-2,31)	

**Desfechos clínicos – n (%)**				
Óbito	3 (9,68)	16 (32)	1,21 (1,16-16,62)	**0,021**

Diálise	0 (0)	3 (6)	–	0,437

LRA: lesão renal aguda; OR: *odds ratio;* IC 95%: intervalo de confiança de 95%; IAM: infarto agudo do miocárdio; IC: insuficiência cardíaca; HAS: hipertensão arterial sistêmica; DM: diabetes melito; DRC: doença renal crônica; pH: potencial hidrogeniônico; IAM: infarto agudo do miocárdio; IC: insuficiência cardíaca; AE: átrio esquerdo; VE: ventrículo esquerdo; DP: desvio-padrão.

Houve semelhança entre as variáveis clínicas avaliadas nos dois grupos, porém o grupo com LRA apresentou predomínio de pacientes com doença renal, bem como níveis mais elevados de ureia e creatinina.

Quanto às características cardiológicas e ecocardiográficas, e a sua relação com o desenvolvimento da injúria renal, a IC foi o principal diagnóstico de admissão entre os pacientes que apresentavam função renal alterada. Não foi encontrada relação entre a redução da fração de ejeção e o desenvolvimento de síndrome cardiorrenal.

Dentro do grupo dos pacientes que não evoluíram com LRA, 9,68% foram a óbito durante a internação hospitalar. Em contrapartida, nos pacientes com LRA, o percentual encontrado foi de 32%, o que mostra uma associação da LRA a mortalidade, *odds ratio* (OR) de 1,21 (intervalo de confiança [IC 95%] entre 1,16 e 16,62) e p = 0,021. No que diz respeito a necessidade de intervenção dialítica, esta só esteve presente em 6% dos pacientes que evoluíram com injúria renal, mas sem diferença entre os grupos.

Os dados apresentados na Tabela 3 mostram que 50 pacientes evoluíram com deterioração da função renal em pelo menos um dos critérios utilizados para a classificação. O KDIGO foi capaz de detectar a injúria renal em 61,73% dos casos. O critério AKIN foi incapaz de detectar a LRA em 14% dos pacientes. Entretanto, não foi observado, no presente estudo, a superioridade do critério KDIGO na detecção precoce das alterações na função renal. A análise multivariada (Tabela 4) mostrou que a LRA foi um fator de risco independente para a mortalidade, com OR ajustado 8,187, IC 95% entre 1,402 e 17,190 e p =0,020.

**Tabela 3:** Incidência de LRA de acordo com os critérios AKIN e KDIGO em pacientes com síndrome cardiorrenal

Variável	AKIN		KDIGO		p – valor
	N	%	N	%	
Estágio 1	35	43,21	39	48,15	0,642
Estágio 2	3	3,7	5	6,17	0,479
Estágio 3	5	6,17	6	7,41	0,763

AKIN: Acute Kidney Injury Network; KDIGO: Kidney Disease: Improving Global Outcomes; LRA: lesão renal aguda.

**Tabela 4:** Análise multivariada de variáveis relacionadas com óbitos na síndrome cardiorrenal.

Variáveis	OR ajustado (IC 95%)	p
Sexo masculino	0,796 (0,241-2,632)	0,708
Idade (+ 1 ano)	1,010 (0,967-1,055)	0,651
HAS	2,228 (0,684-7,261)	0,184
DRC	6,622 (0,901-48,693)	0,063
Ureia (+1 mg/dL)	1,005 (0,983-1,028)	0,660
Lesão renal aguda	8,187 (1,402-17,190)	0,020

OR: Odds ratio; HAS: hipertensão arterial sistêmica; IC 95%: intervalo de confiança de 95%; DRC: doença renal crônica.

## Discussão

Estima-se que mais de 85% da população mundial viva em regiões com baixo e médio desenvolvimento, nas quais é comum a baixa publicação de pesquisas. Fatores socioeconômicos e ambientais, destacando-se a escassez de alimentos, influenciam a evolução da injuria renal aguda das cardiopatias e da síndrome cardiorrenal, conexões habitualmente ignoradas em muitos estudos.^6^^,^^7^ A pesquisa em questão foi desenvolvida em hospital terciário, principal referência no atendimento de emergência de uma população de aproximadamente 1 milhão de pessoas, em um estado do Brasil de baixo desenvolvimento econômico (22^o^ lugar entre 27 unidades da federação no quesito Produto Interno Bruto [PIB]).^6^^,^^7^^,^^11^

No presente estudo, no entanto, identificou-se que as características clínicas e demográficas foram semelhantes às de trabalhos encontrados na literatura, constituindo-se de uma população de idosos predominantemente masculina, como mostrado por Spineti et al.,^3^ com 58% da população estudada deste gênero e média de idade de 63,5 ± 13 anos. Liangos et al.^11^ demonstrou predominância de homens, idosos e DM, HAS e DRC as principais comorbidades associadas.

Trabalhos realizados a partir de pacientes diagnosticados com LRA constataram que as doenças crônicas, principalmente DM e HAS, apresentaram maior associação ao desenvolvimento de LRA.^11^^,^^12^ Entretanto, assim como em nossa pesquisa, o estudo de Caetano et al.^13^ não verificou a associação do desenvolvimento de SCR a antecedentes de IC, DM ou pressão sistólica elevada na admissão, mas à existência de doença renal prévia.

Em um estudo multicêntrico, foram reunidos dados de 105.388 pacientes com IC agudamente descompensada de 274 hospitais dos EUA. Revelou-se que 30% desses pacientes apresentavam também DRC, assim como observado por Damman et al.^14^ em uma metanálise em que a prevalência de DRC foi de 32%. Em nosso estudo, a LRA esteve presente em 61,7% dos pacientes. Essa condição é cada vez mais frequente em pacientes com IC, podendo ser um agravante no que concerne à intensidade dos sintomas, alterando tanto o curso clínico do quadro apresentado como a resposta ao tratamento.^15^Em alguns estudos, a DRC preexistente em pacientes internados por IC descompensada propiciou a evolução à LRA em todos os casos avaliados.^16^

Estudos voltados a avaliar as características laboratoriais mostraram que, na admissão, os pacientes sem síndrome cardiorrenal (SCR) aguda apresentaram valores de ureia, creatinina e potássio mais elevados quando comparados aos sem injúria renal.^3^^,^^13^ Outros trabalhos, além de corroborar com esses dados, demonstraram que pequenas elevações nos valores de creatinina estão significativamente associadas ao aumento da mortalidade em pacientes com LRA.^17^

No presente estudo, a maioria dos pacientes admitidos com IC descompensada apresentavam alguma cardiopatia prévia. Essa característica não foi associada ao desenvolvimento de LRA. Quanto aos parâmetros ecocardiográficos, incluindo a fração de ejeção do ventrículo esquerdo (FEVE), nenhuma característica anatômica ou funcional cardíaca apresentou associação à evolução da SCR. Apesar da LRA ser igualmente prevalente na IC por disfunção sistólica ou diastólica, a injúria renal é geralmente mais grave em pacientes com FEVE reduzida quando comparada aos pacientes com FEVE normal, estando presente em mais de 70% dos casos admitidos em choque cardiogênico.^2^ Resultados semelhantes foram demonstrados em outro estudo, que evidenciou pacientes com IC por disfunção sistólica e FEVE < 40%, presentes em 86% dos casos avaliados com LRA.^3^

Caetano et al.,^13^ através de um estudo que avaliava os parâmetros ecocardiográficos dos pacientes, constataram que a função sistólica estava preservada (FEVE ≥ 50%) em 48,4% dos doentes. Entre os pacientes que desenvolveram LRA, 26 (56,6%) apresentavam comprometimento da fração de ejeção, enquanto um total de 47 (43,1%) pacientes que não apresentavam essa alteração evoluiu com o comprometimento agudo da função renal. No mesmo estudo, demonstrou-se que a insuficiência mitral de moderada a grave esteve presente em 45,1% dos pacientes sem LRA e em 68,4% dos pacientes que desenvolveram a LRA (p = 0,014). Nesse estudo, a FEVE média foi de aproximadamente 36% e apenas três pacientes apresentaram FEVE > 50%, não havendo diferença entre os pacientes com e sem LRA.

Estudos relatam a ocorrência de injúria renal na evolução da IC e sua ocorrência tanto em pacientes com FEVE reduzida quanto nos que apresentavam FEVE normal,^2^^,^^3^^,^^13^ o que reforça a necessidade de avaliação do perfil cardiológico e hemodinâmico durante o curso clínico da piora da função renal. Estudos realizados por Mullens et al.^18^ e Damman et al.^19^ avaliaram o perfil hemodinâmico a partir de métodos invasivos e com terapias intensivas. Dessa forma, sugere-se que a partir da ecocardiografia, que consiste em um método não invasivo, sejam avaliados outros parâmetros que podem ser sugestivos de alterações ligadas a injúria renal, como a avaliação da veia cava, haja vista que os mesmos trabalhos demonstraram correlação entre o aumento da pressão venosa central e a piora da função renal. O diâmetro da veia cava inferior e seu estudo durante a inspiração e expiração são de possível avaliação a partir da ecocardiografia. Entretanto, na literatura, ainda são escassos os dados relacionados com esses parâmetros.

A necessidade de diálise ocorreu nesse estudo em 6% dos pacientes, coincidindo com o observado por Li et al.^20^ em coorte chinesa com 1.005 pacientes (6,4%), e foi semelhante à observada na metanálise de Vandenberghe et al.,^5^ em que a necessidade de terapia renal substitutiva ocorreu em 4,6% nos pacientes com SCR por IC descompensada e em 2,3% na SCR por todas as etiologias. De acordo com Forman et al.,^21^ em pacientes com IC, óbito intra-hospitalar e maior tempo de internação, as complicações ocorrem mais frequentemente em pacientes com LRA. Em nossa pesquisa, observou-se 32% de mortalidade hospitalar em pacientes com LRA, condição em que a análise multivariada representou um fator de risco independente para a mortalidade, OR de 8,187 (1,402-17,190) e p =0,020. Hata et al.,^22^ também em análise retrospectiva de 376 indivíduos admitidos em UTI por IC descompensada, com LRA em 73% da amostra, apurou que esta complicação estava relacionada com elevada mortalidade intra-hospitalar (10,5% *versus* não LRA de 1,0%; p < 0,01) e ao tempo de internação prolongado quando comparados ao grupo-controle. Na análise multivariada da presente pesquisa, a DRC não se mostrou um fator de risco isolado, com OR 6,622 (0,901-48,693) e p =0,063, possivelmente atribuída a pequena amostra. Damman et al.,^14^ em metanálise, encontrou a associação desse fator a mortalidade, OR 2,3 (2,20-2,50) e p < 0,001. Em outro estudo, foi observado uma taxa maior de mortalidade intra-hospitalar dos pacientes que evoluíram com LRA, principalmente entre os que apresentaram maior deterioração da função renal. Em um total de 18 pacientes que foram a óbito, dezessete apresentaram LRA (94,5%), sendo que 76,5% eram classificados como AKIN estágio 3 e 23,5% AKIN estágio 2.^16^

Barros et al.,^16^ em um estudo com 85 pacientes hospitalizados em UTI admitidos com IC descompensada, observaram que 76,5% dos indivíduos durante o período de internação evoluíram com LRA, sendo mais frequente o estágio 3 da classificação AKIN (38,8%), enquanto os estágios 1 e 2 representaram 4,7% e 32,9%, respectivamente. Devemos considerar que, de modo geral, os pacientes internados em UTI apresentam um quadro clínico de maior complexidade, apresentando comprometimento de diversas funções sistêmicas, incluindo a função renal. Dessa forma, é possível que estágios mais elevados nas classificações de LRA sejam encontrados nesse estrato populacional.

De acordo com estudo comparativo entre os critérios RIFLE, AKIN e KDIGO em pacientes no pós-operatório de cirurgia cardíaca, foi demonstrada a superioridade do critério KDIGO com relação aos demais quanto ao seu poder prognóstico nos pacientes.^23^

Em nosso estudo, o critério KDIGO não se mostrou estatisticamente superior ao AKIN, achado semelhante ao de Roy et al.,^24^ que avaliou 637 pacientes com IC e apurou que os critérios AKIN, KDIGO, além de um outro critério proposto pela *Acute Dialysis Quality Initiative* na classificação RIFLE (*Risk, Injury, Failure, Loss e End-Stage Kidney*), se mostraram similares. Em uma grande coorte retrospectiva chinesa, que incluiu pacientes com IC, Li et al.^20^ demonstraram que o KDIGO foi superior aos critérios AKIN e RIFLE. No entanto, a proporção de pacientes que apresentavam estágios 2 e 3 foram superiores aos encontrados em nosso estudo.

### Limitações

Esta pesquisa apresenta algumas limitações, como o fato ter sido realizada em único centro, com número pequeno de pacientes. Além disso, trata-se de um estudo retrospectivo com limitações inerentes a essa modalidade de pesquisa, como a dependência das informações contidas em prontuários médicos. Embora tenha atendido aos requisitos específicos para a modelagem e a seleção de variáveis na regressão logística, não é possível garantir que outras variáveis possam ter contribuído para o resultado apresentado. Da mesma forma, o registro da creatinina não foi diário em todos os casos, e isso poderia interferir na aferição do estágio da LRA. Ademais, o número de pacientes em estágios não iniciais de LRA foi baixo, o que pode ter influenciado a performance dos critérios diagnósticos AKIN e KDIGO.

## Conclusão

Nessa casuística de pacientes admitidos em IC descompensada com cardiopatia prévia ou IAM recente em hospital público terciário de região com baixo desenvolvimento econômico nacional, a ocorrência de LRA foi elevada e constituiu-se em fator de risco independente da taxa de mortalidade. A DRC constituiu-se em fator de risco para o desenvolvimento de LRA. Na população analisada, as alterações cardíacas, anatômicas ou funcionais, avaliadas ao ecocardiograma, não se associaram à ocorrência de LRA. Os critérios diagnósticos KDIGO e AKIN apresentaram performance similar nessa amostragem.

## References

[1] 1. Rangaswami J, Chair V, Bhalla V,Chang TI, Costa S, Lentine KL, et al. Cardiorenal syndrome: classification, pathophysiology, diagnosis and treatment strategies. A scientific statement from the American Heart Association. Circulation. 2019; 139(16): e840-e78. 10.1161/CIR.0000000000000664 30852913

[2] 2. Ronco C, Haapio M, House AA,Anavekar NS, Bellomo R. Cardiorenal syndrome. J Am Coll Cardiol. 2008; 52(19): 1.527-39. 10.1016/j.jacc.2008.07.051 19007588

[3] 3. Spineti PPM, Tedeschi B, Sales ALF. Incidência e preditores de síndrome cardiorrenal aguda durante tratamento de insuficiência cardíaca descompensada: análise de 332 hospitalizações consecutivas. Rev Socerj. 2009; 22(2): 93-8.

[4] 4. Kellum JA, Lameire N, Aspelin P, Barsoum RS, Burdman EA, Goldstein SL, et al. Kidney disease: improving global outcomes (KDIGO) acute kidney injury work group. KDIGO clinical practice guideline for acute kidney injury. Kidney Inter Suppl. 2012; 2: 1-138.

[5] 5. Vandenberghe W, Gevaert S, Kellum JA et al. Acute kidney injury in cardiorenal syndrome type 1 patients: a systematic review and metanalysis. Cardiorenal Med. 2016; 6(2): 116-28. 10.1159/000442300 PMC4789882 26989397

[6] 6. Nascimento GVR, Silva MN, Carvalho Neto JD, Bagshaw SM, Peperstraete H, Herck I, et al. Outcomes in acute kidney injury in noncritically ill patients lately referred to nephrologist in a developing country: a comparison of AKIN and KDIGO criteria. BMC Nephrol. 2020; 21(1), 94. 10.1186/s12882-020-01751-7 PMC7066785 32160876

[7] 7. Banerjee S, Radak T. Association between food insecurity, cardiorenal syndrome and all-cause mortality among low-income adults. Nutrit Health. 2019; 25(4), 245-52. 10.1177/0260106019869069 31464165

[8] 8. Bagshaw SM, George C, Bellomo R. A comparison of the RIFLE and AKIN criteria for acute kidney injury in critically ill patients. Nephrol Dial Transplant. 2008; 23: 1.569-74. 10.1093/ndt/gfn009 18281319

[9] 9. Mehta RL, Kellum JA, Shah SV et al. Acute kidney injury network: report of an initiative to improve outcomes in acute kidney injury. Crit Care. 2007; 11(2): R31. 10.1186/cc5713 PMC2206446 17331245

[10] 10. Instituto Brasileiro de Geografia e Estatistica – IBGE. Produto interno bruto – PIB. Disponível em: https://www.ibge.gov.br/explica/pib.php. Acesso em: 13 maio 2020.

[11] 11. Liangos O, Wald R, O’ Bell JW et al. Epidemiology and outcomes of acute renal failure in hospitalized patients: a national survey. Clin J Am Soc Nephrol. 2006; 1(1): 43-51. 10.2215/CJN.00220605 17699189

[12] 12. Bucuvic EM, Ponce D, Balbi AL. Fatores de risco para mortalidade na lesão renal aguda. Rev. Assoc. Med. Bras. 2011; 57(2): 158-63. 10.1590/s0104-42302011000200012 21537701

[13] 13. Caetano F, Barra S, Faustino A, Botelho A, Mota P, Costa M et al. Cardiorenal syndrome in acute heart failure: a vicious cycle? Rev Port Cardiol. 2014; 33(3): 139-46. 10.1016/j.repc.2013.09.010 24642129

[14] 14. Damman K, Valente MA, Voors AA, O’Connor CM, Velalhuisen DJ, et al. Renal impairment, worsening renal function, and outcome in patients with heart failure: an updated meta-analysis. Eur Heart J; 35(7): 455-69. 10.1093/eurheartj/eht386 24164864

[15] 15. Adams KF Jr, Fonarow GC, Emerman C,L,ejentel T, Costanzo MR, Abraham WT et al. Characteristics and outcomes of patients hospitalized for heart failure in the United States: rationale, design, and preliminary observations from the first 100,000 cases in the Acute Decompensated Heart Failure National Registry (ADHERE). Am Heart J. 2005; 149(2): 209-16. 10.1016/j.ahj.2004.08.005 15846257

[16] 16. Barros LCN, Silveira FS, Silveira MS, Morais TC. Acute kidney injury in hospitalized patients with decompensated heart failure. J Bras Nefrol. 2012; 34(2): 122-9. 10.1590/s0101-28002012000200004 22850913

[17] 17. Chertow GM, Burdick E, Honour M, Bonsentre V, Bates DW. et al. Acute kidney injury, mortality, length of stay and costs in hospitalized patients. J Am Soc Nephrol. 2005; 16(11): 3.365-70. 10.1681/ASN.2004090740 16177006

[18] 18. Mullens W, Abrahams Z, Francis GS, Sokos G, Taylor DO, Starling RC, et al. Importance of venous congestion for worsening of renal function in advanced decompensated heart failure. J Am Coll Cardiol 2009; 53(7): 589-96. 10.1016/j.jacc.2008.05.068 PMC2856960 19215833

[19] 19. Damman K, Deursen VM, Navis G, Voors AA, Veldhuisen D, Hellege HC. Increased central venous pressure is associated with impaired renal function and mortality in a broad spectrum of patients with cardiovascular disease. J Am Coll Cardiol. 2009; 53(7): 582-8. 10.1016/j.jacc.2008.08.080 19215832

[20] 20. Li Z L, Cai L, Liang X,Du Z, Chen Y, An S, et al. Identification and predicting short-term prognosis of early cardiorenal syndrome type 1: KDIGO is superior to RIFLE or AKIN. PLoS One. 2014; 9(12): e114369. 10.1371/journal.pone.0114369 PMC4277271 25542014

[21] 21. Forman DE, Butler J, Wang Y,Abraham WT, O’Connor CM, Gottlieb S, et al. Incidence, predictors at admission and impact of worsening renal function among patients hospitalized with heart failure. J Am Coll Cardiol. 2004; 43(1): 61-7. 10.1016/j.jacc.2003.07.031 14715185

[22] 22. Hata N, Yokoyama S, Shinada T, Koboyashi N, Shirakabe A, Tomita K, et al. Acute kidney injury and outcomes in acute decompensated heart failure: evaluation of the RIFLE criteria in an acutely ill heart failure population. Eur J Heart Fail. 2010; 12(1): 32-7. 10.1093/eurjhf/hfp169 20023042

