# Supplementary material for: Síndrome Cardiorrenal Tipo 1 em Região de Baixo Desenvolvimento: Comparação entre os Critérios AKIN e KDIGO, Necessidade de Diálise e Mortalidade
Source: Arq Bras Cardiol. 2021 Aug 9;117(2):385–91. [Article in Portuguese] doi: 10.36660/abc.20200097 (PMC8395783; doi:10.36660/abc.20200097)
Supplement: Translation

# Translation: Type 1 Cardiorenal Syndrome in Decompensated Heart Failure Patients in a Low-Income Region in Brazil: Incidence of Acute Kidney Injury (AKIN and KDIGO Criteria), Need for Dialysis and Mortality

**Authors:** Ginivaldo Victor Ribeiro do Nascimento, Heitor Carlos Domingues de Brito, Carlos Eduardo Batista de Lima

**Affiliations:** 1 TeresinaPI Brazil Universidade Estadual do Piauí, Teresina, PI - Brazil; 2 TeresinaPI Brazil Faculdade Integral Diferencial Curso de Medicina, Teresina, PI - Brazil; 3 TeresinaPI Brazil Universidade Federal do Piauí, Centro de Ciências da Saúde, Teresina, PI - Brazil; 4 TeresinaPI Brazil Centro de Pesquisa CARDIOLIMA, Teresina, PI - Brazil

**Keywords:** Cardio-Renal Syndrome/complications, Renal Insufficiency, Acute Kidney, Injury/standards(AKIN), Kidney Disease Improving Global Outcome/standards(KDIGO)

## Abstract

**Background:**

Type 1 cardiorenal syndrome is associated with higher mortality in heart failure patients. However, few studies have compared the diagnostic criteria of acute kidney injury (AKI) in this population.

**Objective:**

To assess clinical and functional features and factors associated AKI in patients with heart failure.

**Method:**

Retrospective, cohort study on patients with decompensated heart failure or recent acute myocardial infarction, conducted in a tertiary hospital in a low-income region of Brazil. Clinical, laboratory and echocardiographic features were compared between patients with and without AKI according to the Acute Kidney Network (AKIN) and Kidney Disease: Improving Global Outcomes (KDIGO) criteria. The level of statistical significance was set at p < 0.05.

**Results:**

Of 81 patients, 61.73% had AKI. Mean creatinine and urea levels were 1.79±1.0 mg/dL and 81.5±46.0 mg/dL, respectively, and higher in the group with AKI (p < 0.05). No evidence of a relationship between cardiac changes and reduced renal function. Chronic renal disease was associated with higher prevalence of AKI. Higher mortality was observed in patients with AKI than in patients without AKI (32.0% vs. 9.8%, p = 0.04, OR 8.187 ad 95% confidence interval 1.402-17.190, p = 0.020).

**Conclusion:**

In this population of patients with heart failure, AKI was highly prevalent and considered an independent risk factor for mortality. Cardiac changes were not associated with AKI, and the KDIGO and AKIN criteria showed similar performance.

## Introduction

Cardiorenal syndrome (CRS) encompasses various acute and chronic conditions in which dysfunction in one organ (either the heart or kidneys) implicates dysfunction in the other.^1^^,^^2^ Approximately one third of patients with heart failure (HF) decompensation may also develop acute renal function impairment, which is characterized as type 1 CRS.^3^

Ventricular dysfunction can cause negative effects on renal function and, meanwhile, renal insufficiency may significantly impair cardiac function. Direct and indirect effects of each dysfunctional organ can initiate and mutually perpetuate a combined set of disorders.^2^

In the last decades, the term “acute kidney injury” (AKI) has been revised, with emphasis on a progressive pathophysiological process that may be noticeable by the presence of small changes in markers of renal injury, especially creatinine. On this regard, two diagnostic criteria have been proposed, the Acute Kidney Network (AKIN) and the Kidney Disease: Improving Global Outcomes (KDIGO).^1^^,^^4^The former, developed to harmonize previous definitions and criteria, distinguish from the term “worsening of renal function”, and have been widely used in investigations of AKI and CRS.^1^^,^^4^^,^^5^ Few studies in the literature have evaluated these AKI and CRS criteria, particularly in emerging countries and low index of economic development.^6^^,^^7^

The aim of the present study was to compare the incidence of CRS using the AKIN and the KDIGO criteria, and to assess risk factors for CRS, need for dialysis, and mortality in patients with decompensated HF.

## Methods

This was an observational, retrospective, cohort study conducted in a tertiary referral hospital for urgency and emergency care in Teresina, Brazil – the *Hospital de Urgências de Teresina*. All patients with a history of heart disease admitted with decompensated HF, and patients with recent acute myocardial infarction (AMI) with echocardiographic evidence of reduced ejection fraction (<55%) were included in the study. Patients younger than 18 years, kidney transplanted patients, patients on chronic dialysis and those without at least two creatinine measurements performed during hospitalization were excluded.

This study was reviewed and approved by the local ethics committee (CAAE n. 54207914.5.0000.5211). Data were obtained by reviewing medical records and echocardiographic findings of patients hospitalized from 01 January to 31 December 2014. The variables studied included admission date and diagnosis, age, sex, comorbidities –systemic arterial hypertension (SAH), diabetes mellitus (DM), dilated cardiomyopathy, cerebrovascular disease, chronic renal disease (CRD), liver disease – laboratory parameters (creatinine, urea, potassium, bicarbonate, pH), clinical improvement, hospital discharge and death.

AKI was defined based on changes in creatinine levels, following the two diagnostic criteria (AKIN and KDIGO). The AKIN proposes a diagnosis of AKI based on change between two creatinine values within a 48-hour period, and need for renal replacement therapy. An absolute increase in serum creatinine greater than 0.3 mg/dL or a 1.5-1.9 times baseline is classified as stage 1 AKI. An increase 2.0–3.0 times baseline is classified as stage 2 AKI. Patients with increase in serum creatinine 3.0 times baseline or serum creatinine levels equal to or greater than 4.0 mg/dl (abrupt rise of at least 0.5 mg/dL) or initiation of renal replacement therapy are classified as stage 3 AKI.^8^^,^^9^

According to the KDIGO criteria, an increase in serum creatine 1.5 times baseline within seven days or an increase by 0.3 mg/dL within 48 hours is classified as stage 1, an increase equal to or greater than 2 times baseline is classified as stage 2, and stage 3 is considered an increase in serum creatinine 3 times baseline (to ≥4 mg/dL) or on renal replacement therapy.^4^

### Statistical analysis

Continuous variables were expressed as mean and standard deviation, according to normality of data distribution verified by the Kolmogorov-Smirnov test, and compared using the unpaired Student’s t-test. Categorical variables were expressed as proportions and compared by Pearson chi-square test. The combined effect of predictive variables on the response variable was assessed using multiple logistic regression models with adjusted odds ratio (OR). The variables that showed a trend for an association (p<0.2) in the bivariate analysis were added in the regression model, and those with statistically significance association (p< 0.05) were maintained in the model. The final multiple logistic regression model was adjusted using the Enter model. The Hosmer–Lemeshow test, a statistical test for goodness of fit for logistic regression models, showed that the final model was adequate to explain the response variable. The variance inflation factor (VIF) was used to assess multicollinearity among the independent variables, and a VIF cutoff of 4 was adopted to identify multicollinearity. However, the test did not detect multicollinearity among the variables studied. A significance level of 5% was set for all the statistical tests. Data were analyzed using the R-Project software, version 3.0.2, and the Statistical Package for the Social Science (SPSS), version 20.0.

## Results

A total of 81 patients admitted for compensated HF or recent AMI were included in the study. Clinical and demographic characteristics of patients are described in Table 1. Mean age of patients was 67.02 ± 14.97 years, and 43 patients (53.1%) were men. The diagnosis of decompensated HF was more common in patients with previous heart disease, and SAH was the most common comorbidity.

**Table 1:** General characteristics of patients with heart failure (ischemic and non-ischemic) evaluated for the presence of acute kidney injury (n = 81)

Variable	N
**Age (years)**	67.02 ± 14.97
Mean ± SD	
**Sex (%)**	>43 (53.09)
Male	
Diagnosis (%)		
Recent AMI	16 (19.75)
HF with a history of heart disease	62 (76.55)
History of HF and recent AMI	3 (3.7)
Other diagnoses*	8 (9.88)
**Comorbidities (%)**		
SAH	48 (59.26)
DM	26 (32.1)
CRD	20 (24.69)
Cerebrovascular disease	7 (8.64)
Liver disease	6 (7.41)
Others	24 (29.63)

AMI: acute myocardial infarction; HF: heart failure; SAH: systemic arterial hypertension; DM: diabetes mellitus; CRD: chronic renal disease; SD: standard deviation.

* Patients with decompensated heart failure and left ventricular ejection fraction < 55% without a history of heart disease or recent acute myocardial, arrhythmias, hypertensive pulmonary edema or infections.

The study population was divided into three subgroups, according to the presence or not of AKI. Clinical, laboratory and echocardiographic features of these patients (50 with AKI and 32 without AKI) are described in Table 2. Although no differences in clinical variables were observed between the two groups, most patients with AKI had renal disease and elevated levels of urea and creatinine.

**Table 2:** Clinical, laboratory and echocardiographic features, and clinical outcomes of patients with and without acute kidney injury of hospitalized patients with heart failure or acute myocardial infarction (n = 81)

Variables	Without AKI (n = 31)	With AKI (n = 50)	OR (95%CI)	p – value
**Clinical data**					
Age (± SD)	64.03 ± 16.08	68.88 ± 14.08	1.02 (0.99-1.05)	0.172

Male sex – n (%)	20 (64.52)	23 (46)	2.13 (0.85-5.37)	0.104

SAH – n (%)	15 (48.39)	33 (66)	0.48 (0.20-1.20)	0.116
DM – n (%)	8 (25.81)	18 (36)	0.62 (0.23-1.66)	0.339
CRD – n (%)	1 (3.23)	19 (38)	18.4 (2.31-146.10)	**0.001**
Cerebrovascular disease - n (%)	1 (3.23)	6 (12)	4.09 (0.47-35.73)	0.337
Liver disease – n (%)	3 (9.68)	3 (6)	0.60 (0.11-3.16)	0.858
Others– n (%)	7 (22.58)	17 (34)	–	–

Laboratory data (± DP)					
Urea (mg/dL)	46.65 ± 25.66	81.52 ± 46.04	1.03 (1.01-1.05)	**0.001**
Creatinine (mg/dL)	1.17 ± 0.76	1.79 ± 0.97	1.02 (1.01-1.04)	**0.002**
Potassium (mEq/L)	4.11 ± 0.75	4.4 ± 1.02	2.76 (1.29-5.91)	0.155
Bicarbonate	24.12 ± 6.11	22.01 ± 4.71	0.92 (0.79-1.07)	0.355
pH	7.417 ± 0.05	7.374 ± 0.1	0.01 (0.01-74.88)	0.069

Echocardiographic features					
Recent AMI – n (%)	8 (25.8)	8 (16)	0.61 (0.22-1.73)	0.507
HF with previous heart disease – n (%)	22 (70.97)	40 (80)	1.83 (0.61-5.51)	0.429
History of HF and recent AMI – n (%)	1 (3.23)	2 (4)	-	0.999

Ejection fraction: % (± DP)	35.86 ± 10.79	36.09 ± 10.79	1.01 (0.97-1.05)	0.598

Left atrial diameter: mm (± DP)	39.51 ± 7.45	39.51 ± 7.45	1.02 (0.96 - 1.08)	0.624

Myocardial thickness – n (%)					
Increased	16 (51.61)	21 (42)	0.77 (0.31-1.90)	0.538
Normal	15 (48.39)	29 (58)	Reference	

LF systolic dysfunction – n (%)					
Mild	1 (3.23)	2 (4)	Reference	0.999
Moderate/severe	30 (96.77)	48 (96)	1.25 (0.18-2.31)	

Clinical outcomes – n (%)					
Death	3 (9.68)	16 (32)	1.21 (1.16-16.62)	**0.021**

Dialysis	0 (0)	3 (6)	–	0.437

AKI: acute kidney injury; OR: odds ratio; 95% CI: 95% confidence interval; AMI: acute myocardial infarction; HF: heart failure; SAH: systemic arterial hypertension; DM: diabetes mellitus; CRD: chronic renal disease; AMI: acute myocardial infarction; LV: left ventricular; SD: standard deviation.

Regarding the association of cardiac and echocardiographic features with development of kidney injury, HF was the main admission diagnosis among patients with altered renal function. No relationship was found between reduced ejection fraction and development of CRS.

With respect to in-hospital mortality, while a 9.7% rate was found in patients without AKI, 32% of patients with AKI died during hospitalization, indicating an association of AKI with mortality (OR 1.21, 95% confidence interval [95% CI] between 1.16 and 16.62, p = 0.021). The need for dialysis was observed in only 6% of patients with AKI, but without statistically significant difference between the groups.

Deterioration of renal function was observed in 50 patients according to at least one diagnostic criteria (KDIGO and AKIN). Using the KDIGO criteria, kidney injury was detected in 61.7% of patients, whereas the AKIN was unable to detect AKI in 14% of patients (Table 3). However, in the present study, the KDIGO criteria was not superior to AKIN in detecting early changes in renal function. Multivariate analysis (Table 4) showed that AKI was an independent risk factor of mortality, with an adjusted OR of 8.187, 95%CI 1.402-17.190, and p=0.020.

**Table 3:** Incidence and staging of acute kidney injury according to the diagnostic criteria proposed by the Acute Kidney Injury Network (AKIN) and the Kidney Disease: Improving Global Outcomes (KDIGO) in patients with cardiorenal syndrome

AKI staging	AKIN		KDIGO		p – value
	N	%	N	%	
Stage 1	35	43.21	39	48.15	0.642
Stage 2	3	3.7	5	6.17	0.479
Stage 3	5	6.17	6	7.41	0.763

AKI: acute kidney injury.

**Table 4:** Multivariate analysis of variables related to cardiorenal syndrome in hospitalized patients with decompensated heart failure or recent acute myocardial infarction (n=81)

Variables	Adjusted OR (95%CI)	p
Male sex	0.796 (0.241-2.632)	0.708
Age (+ 1 year)	1.010 (0.967-1.055)	0.651
Systemic arterial hypertension	2.228 (0.684-7.261)	0.184
Chronic renal disease	6.622 (0.901-48.693)	0.063
Urea (+1 mg/dL)	1.005 (0.983-1.028)	0.660
Acute kidney injury	8.187 (1.402-17.190)	0.020

OR: Odds ratio;95%CI: 95% confidence interval.

## Discussion

It is estimated that more than 85% of the world population live in low/medium income countries, where the development of scientific studies is typically low. Socioeconomic and environmental factors, including food shortage, affect the outcome of AKI in heart diseases and CRS, and such associations are frequently ignored in many studies.^6^^,^^7^ The present study was conducted in a tertiary hospital, the main emergency referral center of a population of nearly one million people, in a state of low economic development (ranking 22^nd^ of 27 federative units in terms of gross domestic product) in Brazil.^6^^,^^7^^,^^11^

However, in the study population, clinical and demographic features were similar to those reported in the literature, with a predominantly male, older patients, as reported by Spineti et al.,^3^ where 58% of the sample were men, mean age of 63.5 ± 13 years. Liangos et al.^11^ also shown a predominance of male, older patients, and DM, SAH, and CRD as the main comorbidities.

Studies on patients diagnosed with AKI have demonstrated that chronic conditions, especially DM and SAH, are more strongly associated with AKI.^11^^,^^12^ However, similar to our study, Caetano et al.^13^ did not find an association of CRS with a history of HF, DM, or elevated blood pressure at hospital admission, but with history of kidney disease.

In a multicenter study, data of 105,388 patients with acutely decompensated HF were collected from 274 hospitals in the USA. The prevalence of CRS in this population was 30%, which was similar to that (32%) reported in the meta-analysis by Damman et al.^14^ In our study, AKI was present in 61.7% of patients. This is condition, more and more common in HF patients, can be an aggravating factor for symptom severity, and change not only the clinical course of disease, but also response to treatment.^15^ In some studies, preexisting CRD in patients admitted for decompensated HF was associated with development of AKI in all cases.^16^

Analyses of laboratory data of patients admitted with acute CRS had higher values of urea, creatinine and potassium compared with those without renal injury.^3^^,^^13^ Other studies not only corroborated these findings, but also showed that small changes in creatinine levels are significantly associated with an increase in mortality in patients with AKI.^17^

In the present study, most patients admitted for decompensated HF had a history of heart disease, and this factor was not associated with the development of AKI. Regarding the echocardiographic parameters, including left ventricular ejection fraction (LVEF), no cardiac structural or functional parameter was associated with the course of CRS. Although AKI is equally prevalent in HF due to systolic dysfunction and diastolic dysfunction, kidney injury is generally more severe in patients with reduced LVEF as compared with those with normal LVEF, and found in more than 70% of patients admitted with cardiogenic shock.^2^ Similar findings were reported in another study that showed that 86% of patients with AKI had HF and LVEF<40%.^3^

Caetano et al.,^13^ in an echocardiographic study of patients with CRS, showed that 48.4% of patients had preserved systolic function (LVEF ≥ 50%). Among the patients with AKI, 26 (56.6%) had compromised ejection fraction, whereas 47 (43.1%) of patients without AKI had acute renal dysfunction. In the same study,^13^ moderate or severe mitral insufficiency was found in 68.4% and 45.1% of patients with and without AKI, respectively (p=0.014). In addition, mean LVEF was approximately 36% and only three patients had a LVEF > 50%, with no difference between patients with and without AKI.

Studies have reported the occurrence of AKI in HF in patients with both reduced and preserved LVEF,^2^^,^^3^^,^^13^ which reinforces the need for evaluating the cardiac and hemodynamic function in patients with worsening renal function. Studies by Mullens et al.^18^ and Damman et al.^19^ evaluated the hemodynamic profile of patients with cardiovascular disease using invasive methods and intensive therapies. Thus, it is suggested that other parameters, indicative of renal injury, such as changes in the vena cava, could be evaluated by echocardiography, a non-invasive method, since these same studies have reported a correlation between increased central venous pressure and worsening of renal function. Although the assessment of inferior vena cava diameter during inspiration and expiration is possible by echocardiography, few studies have investigated these parameters.

Need for dialysis was seen in 6% of our patients, which is in accordance with the study by Li et al.,^20^ with a cohort of 1,005 Chinese patients (6.4%). Also, the meta-analysis by Vandenberghe et al.^5^ showed a need for renal replacement therapy in 4.6% of patients with CRS due to decompensated HF and 2.3% of patients with CRS due to other causes. According to Forman et al.,^21^ in HF patients with longer hospitalizations and in-hospital death, complications are more common in patients with AKI. In our study, we observed a 32% mortality rate in patients with AKI, which represented an independent risk factor for mortality (OR 8.187 [1.402 – 17.190], p=0.020). Hata et al.,^22^ in a retrospective analysis of 376 patients admitted to the intensive care unit (ICU) with decompensated HF, AKI was detected in 73% of patients and was correlated with high in-hospital mortality (10.5% versus 1.0% in non-AKI patients; p<0.01), and longer hospital stay as compared with the control group. In our study, CRS alone was not considered a risk factor by the multivariate analysis (OR 6.622 [0.901-48.693], p=0.063), possibly because of the small sample size. However, Damman et al.^14^ reported in a meta-analysis, a significant association between CRS and mortality (2.3 [2.20-2.50], p < 0.001). In another study, higher in-hospital mortality was found in patients with AKI, especially among those who had greater worsening of renal function. Of 18 patients who died, 17 (94.5%) had AKI, 76.5% AKIN stage 3 and 23.5% AKIN stage 2.^16^

Barros et al.,^16^ in a study with 85 hospitalized patients admitted to the ICU with decompensated HF, found that 76,5% of patients had AKI, mainly at stage 3 (38.8%) (AKIN criteria), followed by stage 2 (32.9%) and stage 1 (4.7%). We should consider that, in general, critically ill patients in ICUs have impairment of many organs, including renal dysfunction. Therefore, it is possible that patients with AKI at more advanced stages may be found in this population.

According to a comparative study between RIFLE (Risk, Injury, Failure, Loss and End-Stage Kidney, classification proposed by the Acute Dialysis Quality Initiative group), AKIN and KDIGO in patients in the post-operative period of cardiac surgery, the prognostic power of the KDIGO criteria was superior to both AKIN and RIFLE.^23^ In our study, however, KDIGO was not superior to AKIN, which was similar to the findings reported by Roy et al.,^24^ who evaluated 637 patients and found a similar performance of AKIN, KDIGO and RIFLE. In a large Chinese retrospective, cohort study, that included patients with HF, Li et al.^20^ showed that KDIGO was superior to both AKIN and RIFLE, but the proportions of patients with AKI at stages 2 and 3 were higher than in our study.

### Limitations

This study has some limitations that need to be considered. First, this was a single-center study, involving a small number of patients. Second, there were limitations inherent to the retrospective design of the study, including reliance on medical records. Although all specific requirements for modeling and variable selection of logistic regression were met, the possibility that other variables influenced the results cannot be excluded. Likewise, creatinine measurements were not recorded daily, which may have affected the evaluation of AKI staging. Finally, the small number of patients with AKI at initial stages may have influenced the performance of the AKIN and KDIGO diagnostic criteria.

## Conclusion

In this population of patients admitted to a public tertiary hospital of a low-income region, with decompensated HF and a history of heart disease or recent AMI, there was a high occurrence of AKI, which was an independent risk factor for mortality. CRS was a risk factor for AKI. In addition, cardiac structural and functional changes, evaluated by echocardiography, were not associated with the development of AKI. The KDIGO and AKIN diagnostic criteria showed similar performance in this population.
